# Correspondence of Yolk Sac and Embryonic Genotypes in F0 Mouse CRISPants

**DOI:** 10.18103/mra.v11i6.3989

**Published:** 2023-06-26

**Authors:** Kayla T. B. Fuselier, Claudia Kruger, J. Michael Salbaum, Claudia Kappen

**Affiliations:** 1 Department of Developmental Biology, Pennington Biomedical Research Center/Louisiana State University System, 6400 Perkins Road, Baton Rouge, LA 70808, USA; 2 Department of Regulation of Gene Expression, Pennington Biomedical Research Center/Louisiana State University System, 6400 Perkins Road, Baton Rouge, LA 70808, USA

## Abstract

CRISPR-mediated genome editing *in vivo* can be accompanied by prolonged stability of the Cas9 protein in mouse embryos. Then, genome edited variant alleles will be induced as long as Cas9 protein is active, and unmodified wildtype target loci are available. The corollary is that CRISPR-modified alleles that arise after the first zygotic cell division potentially could be distributed asymmetrically to the cell lineages that are specified early during morula and blastocyst development. This has practical implications for the investigation of F0 generation individuals, as cells in embryonic and extraembryonic tissues, such as the visceral yolk sac, might end up inheriting different genotypes. We here investigated the hypothetically possible scenarios by genotyping individual F0 CRISPants and their associated visceral yolk sacs in parallel. In all cases, we found that embryonic genotype was accurately reflected by yolk sac genotyping, with the two tissues indicating genetic congruence, even when the conceptus was a mosaic of cells with distinct allele configurations. Nevertheless, low abundance of a variant allele may represent a private mutation occurring only in the yolk sac, and in those rare cases, additional genotyping to determine the mutational status of the embryo proper is warranted.

## Introduction

The ability to target genome editing events to specific sites and the relative speed of application has brought CRISPR technology to the forefront for genetic manipulation in a wide variety of species^1^. In its simplest instantiation, the CRISPR approach produces DNA breaks at a designated cut site theta, that the host cell then repairs through homology-directed repair (HDR) based on a template, or through non-homologous end-joining (NHEJ)^2^. In addition to its ease of use, a particular promise of the CRISPR technology lies in the simultaneous targeting of multiple gene loci^3–5^, to create genetic conditions that allow investigation of gene-gene-interactions without extensive and sometimes complicated breeding strategies^6^.

However, widespread use in the past years has also revealed weaknesses of the CRISPR technology, such as the potential for creating off-target events^7^, or undesired inaccuracies at the intended targeted locus^8,9^. In approaches designed to rely on the repair of CRISPR/Cas9-induced DNA double strand breaks by non-homologous end-joining (NHEJ), small deletions of up to 10 base pairs can be expected at the target cut site theta^10^. However, we previously discovered that a single guide RNA targeted to the mouse T/brachyury gene, when co-injected with Cas9 protein into fertilized mouse oocytes, can generate numerous deletion variants with breakpoints even far away from the target site theta, and of varying length and direction^11^.

In addition, we detected multiple variant alleles within the same animal, implying that the first Cas9-guideRNA-induced DNA break occurred only on one chromosome, and that additional mutations were induced during further cell division(s). Since any diploid cell can only contain two alleles of any given gene, any individual with 3 or more distinct alleles has to be a mosaic, composed of cells that contain distinct CRISPR induced mutant alleles^11^.

Such a situation presents a particular complexity for the investigation of F0 generation individuals, particularly at embryonic or fetal stages, because specification of the inner cell mass -which gives rise to the embryo proper- occurs as early as the 16-cell morula stage, and by the 32-cell stage, the first primitive endoderm cells are detectable^12^ that contribute to the visceral yolk sac after implantation^13^. Examination of F0 individuals becomes necessary when the targeted gene of interest is suspected or known to cause lethality at embryonic or fetal stages^14^; in those approaches, the associated visceral yolk sac is typically used for genotyping. Because the progenitors of visceral yolk sac endoderm are specified by the early blastocyst stage, it is theoretically possible that the yolk sac contains different variant alleles than the embryo proper. In a scenario with multiple consecutive CRISPR-induced mutation events, the yolk sac then may or may not inform appropriately about embryonic genotype of individual F0 CRISPants.

In the present study, we conducted explicit tests for this possibility, by genotyping both embryo and yolk sac from the same conceptus at midgestation, using a validated guide RNA directed to the same target site in the T/brachyury locus, published previously^11^. This guide RNA targets exon 3, which encodes a part of the DNA-binding domain of the T transcription factor. We showed that disruptions of this exon by CRISPR-induced deletions produce similar morphological anomalies as observed in conventional T mutant embryos^11^: a wide variety of defects in mesoderm derivatives, tails that are shorter, curled or absent, abnormal neural tube closure, kinked or wavy neural tubes^15^.

These phenotypes are also observed in our newly generated independent F0 CRISPants, which recapitulate some gene editing events identified in our previous study^11^, but also contained distinct new alleles. Moreover, we failed to detect unique mutant alleles in the embryo that weren’t also present in the yolk sac. Taken together, our present results indicate that yolk sac genotyping appropriately reflects embryonic genotype in F0 mouse CRISPants.

## Materials and Methods

### Animals

All mice (Charles River Laboratories International, Inc.) were housed in individually vented cages in a 7-hour dark/light cycle. Female mice of the FVB/N mouse strain^16^ were quarantined for two weeks after arrival and fed a Purina Chow diet. These mice were used as zygote donors and super ovulated by intraperitoneal injection of 5 IU PMSG (ProSpec-Tany TechnoGene Ltd) one hour past the middle of the light cycle two days before breeding. One hour before matings to FVB/N males were set up, females were injected with 5 IU Human Chorionic Gonadotropin (ProSpec-Tany TechnoGene Ltd). If copulation plugs were detected the next morning, this was counted as day E0.5 of gestation.

Females of the outbred CD-1 mouse strain were used as foster dams^17^ and fed a Purina Breeder Chow diet (LabDiet 5015). They were used as surrogates at >than 6 weeks of age, after mating with a vasectomized CD-1 male to induce pseudopregnancy.

All animal husbandry was carried out in strict accordance with the recommendations in the Guide for the Care and Use of Laboratory Animals of the National Institute of Health of the United States of America, covered by protocols approved by the Institutional Animal Care and Use Committee of the Pennington Biomedical Research Center.

### Microinjection and transfer

Fertilized oocytes at the single-cell stage were harvested from FVB/N females on E0.5 and cultured in EmbryoMax^®^ Advanced KSOM Embryo Medium (EMD Millipore) at 37°C in an atmosphere containing 5% CO_2_ before and after microinjection. Microinjections were performed as previously described^11^ and consisted of 25 ng/μl TrueGuide^™^ sgRNA and 50 ng/μl TrueCut^™^ Cas9 Protein v2 (Invitrogen) diluted in EmbryoMax^®^ Electroporation Buffer (EMD Millipore). The sgRNA targeting T/brachyury exon 3 was the same as in our earlier publication^11^. A maximum of 28 injected zygotes were transferred bilaterally into the infundibulum of the uterine horns of pseudopregnant CD-1 females. Surrogate females were euthanized at E10.5, and conceptuses were recovered for morphological analysis and genotyping.

### Dissections

Conceptuses were isolated at E10.5 within their individual decidua, and placed in separate fresh dishes of OptiMEM (Invitrogen) for dissection. Embryos and their corresponding visceral yolk sacs were dissected apart and kept as individual samples for genotype analysis. Imaging of embryos was performed using a Leica S9i stereo microscope, and documented using V4.12.0 Leica Application Suite software (Leica Microsystems Co.). Samples were rinsed in 500μl sterile PBS and stored in 100μl PBS at −20°C until genomic DNA isolation.

### DNA isolation and PCR amplification

Stored samples were equilibrated to room temperature. To each sample 100μl of 2X lysis buffer (1X: 50 mM Tris pH8.0, 100 mM EDTA, 100 mM NaCl, 1 % SDS) was added. Proteinase K digestion (10μl of a 10 mg/ml stock solution) was performed at 55°C overnight using a Thermomixer R (Eppendorf) set at 750 rpm. The next morning, 10μl 8M LiCl, 1μl Glycogen and 200μl Isopropanol was added to each sample, vortexed and incubated for 30min at −20°C. After centrifugation (Eppendorf Centrifuge 5417R) at 14000rpm for 30 minutes at 4°C supernatant was discarded and the pellet washed with 70% Ethanol. All Ethanol was removed after another centrifugation step and the pellet was air-dried for 15 minutes. DNA pellets were resuspended in 20μl LoTE buffer (10mM TrisCl pH8, 0.01mM EDTA) and incubated for 15 minutes at 55°C in a Thermomixer. DNA concentrations were determined using the Qubit dsDNA high sensitivity kit on the Qubit fluorometer instrument (Invitrogen).

PCR amplification was performed in 50μl reactions using a T100 Thermal Cycler (BioRad). The PCR primer pair was ordered from IDT (Integrated DNA Technologies). Reactions consisted of 2μl gDNA (25 ng/μl), 2μl forward primer (5’-CAGAGGTTCTCACCGAGAGG-3’, 10μM), 2μl reverse primer (5’-GCTGGCGTTATGACTCACAG-3’, 10μM), 19μl water and 25μl Long Amp Taq 2x master mix (New England BioLabs). Cycling conditions were 3 minutes at 95 °C, followed by 30 cycles of 15 seconds melting at 95 °C, 20 seconds annealing at 59 °C, 6 minutes and 30 seconds extension at 65 °C, with a final step of 10 minutes at 65 °C and a hold at 10 °C. The amplicons (6.58 kb) were purified with the QIAquick PCR column purification Kit (Quiagen) according to the manufacturer’s manual. Concentrations were determined using the NanoDrop One instrument (Thermo Scientific).

### Library preparations

Libraries were generated using the PCR barcoding (96) genomic DNA Kit (SQK-LSK109) and sequenced on the MinION Portable Sequencer (Oxford Nanopore Technologies)^18^. Input DNA ends were prepared for barcode adapter attachment followed by a 17-cycle PCR amplification using barcoded primers and LongAmp Taq 2x master mix (New England BioLabs). Samples were pooled, DNA ends prepared for sequencing adapter ligation and loaded into the flow cell. All DNA purification steps were carried out with AMPure XP beads (Agencourt). The MinKNOW 21.11.8 version software was used for base-calling at high accuracy setting and created fastq files, which were merged for every sample before submitting the data to CRISPresso2 software for analysis.

### Analysis of sequencing data

The CRISPResso version 2.2.9 software^19^ was used to analyze obtained CRISPR edited sequences for the T/Brachyury gene, using default settings and Crispresso1 mode. Sequences of the sgRNA (ACTCTCACGATGTGAATCCG) as well as PCR amplicons were provided for CRISPResso analyses.

## Results

We previously showed that editing of the T/brachyury gene by CRISPR methodology results in a wide spectrum of deletions that were only accurately detected by long-read single molecule DNA sequencing. Here, using the same methodology, we sought to determine, for new independently generated CRISPants, to what extent CRISPR-induced gene edits in the yolk sac can accurately inform about the embryonic genotype. To this end, fertilized oocytes of the FVB/N strain were microinjected with single guide RNA and Cas9 protein, and transferred into the oviducts of pseudo-pregnant recipient females of the CD-1 strain that served as surrogates. Having performed multiple injection sessions, we report here on a single such experiment, from which we recovered 10 successfully implanted conceptuses at day E10.5 (counted relative to the day of donor oocyte harvest). Yolk sacs were dissected away from the embryos proper, and the samples originating from the same conceptus were processed in parallel. Embryos were inspected for microscopically visible malformations, which were documented by digital imaging. Then, genomic DNA was prepared from all visceral yolk sac and embryonic samples, and subjected to PCR amplification and long-read single-molecule DNA sequencing of amplicons as described previously^11^.

**Figure 1:**
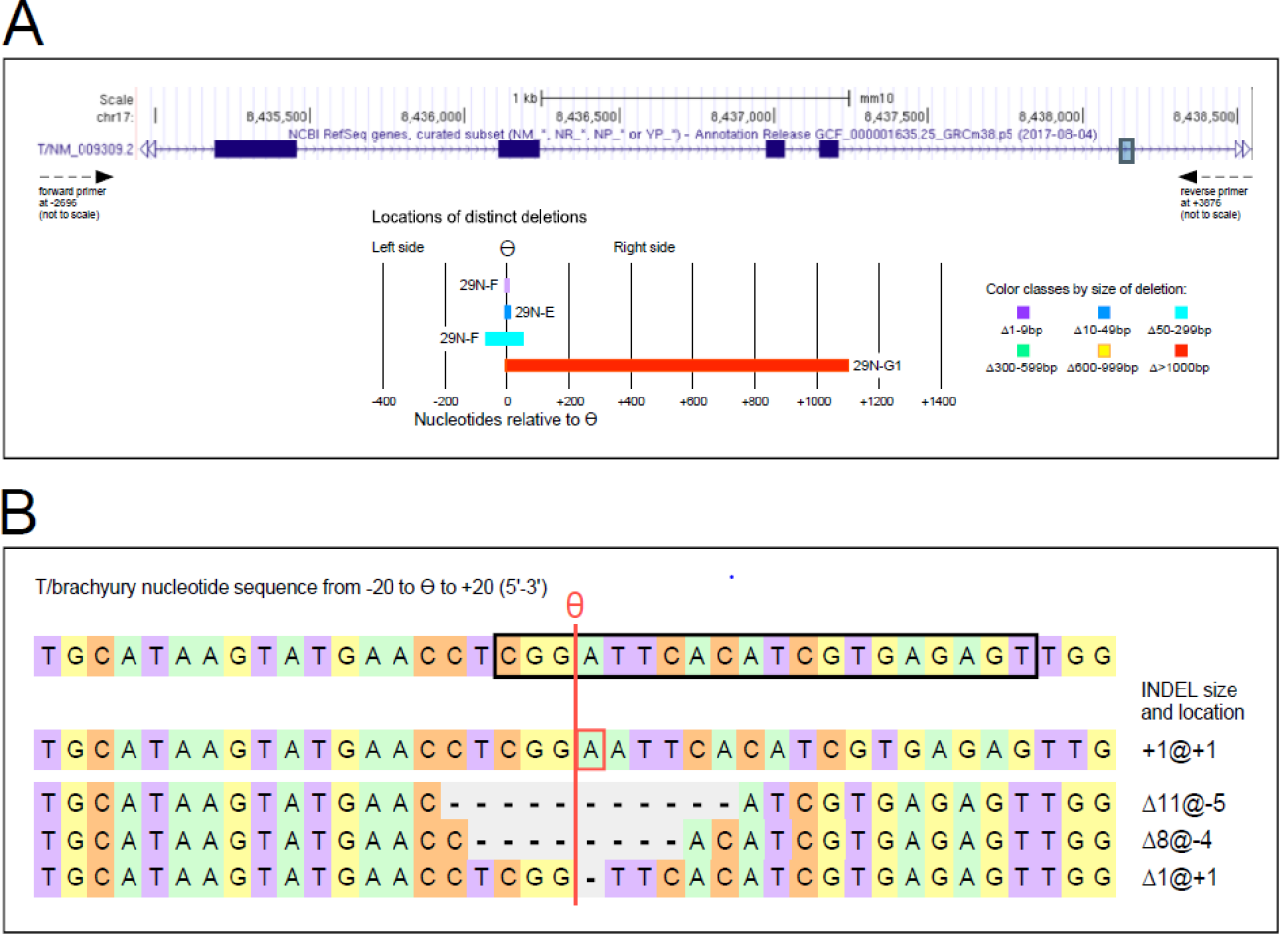
CRISPR-induced mutations in the T/brachyury gene The structure of the T gene is shown in Panel A, as depicted on the NCBI website. The lengths and locations of the 4 longer deletions (of increasing sizes) are depicted in different colors, with the position of theta ϴ and sample designations indicated. Panel B shows the nucleotide sequences of the smaller INDELs around ϴ, within 20 bp on either side. The sequence complementary to the guide RNA is contained within the black box frame. A red vertical line denotes the position of ϴ.

We identified variant alleles in 4 yolk sac/embryo sample pairs, while the other conceptuses yielded only wildtype sequences, as shown in Table 1. Collectively, 6 distinct variant sequences were detected at the T/brachyury locus: a one base pair insertion at the predicted cut site theta ϴ (ins: +1bp@1), a 1 base pair deletion at ϴ (Δ1bp@1), a deletion of 8 base pairs starting at −4 relative to ϴ (Δ8bp@-4), and larger deletions that all originate at different positions relative to ϴ (Δ11bp@-5; Δ119bp@-68; and Δ1093bp@-2). The deletions are shown in Figure 1, where Panel A depicts the region of the T/brachyury gene covered by each deletion, and Panel B displays sequences with edits around ϴ, including the 1bp insertion that occurred independently in individuals 29N-A and 29N-E.

**Figure 2:**
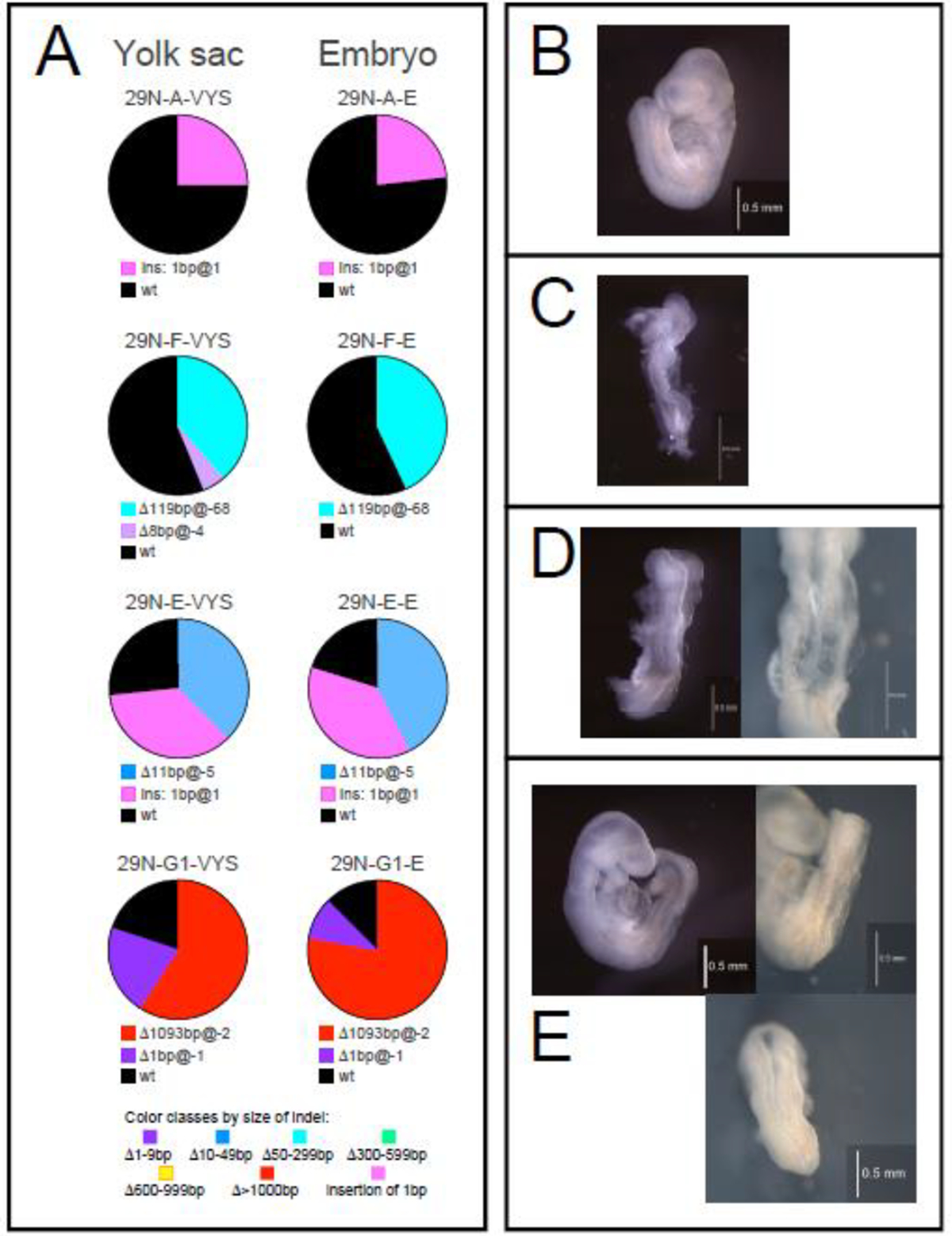
Mutant alleles in yolk sac and embryo pairs, and corresponding embryo morphology Fertilized mouse oocytes were microinjected with guide RNA and Cas9 protein, and transferred into pseudo-pregnant foster dams. Conceptuses were recovered at day E10.5, visceral yolk sac was dissected away, and the embryo appearance was inspected under a stereo microscope before samples were processed for genomic DNA preparation and sequencing. Panel A displays the relative representation of each distinct allele in a pie chart for each sample, consistent with the color scheme in Figure 1; wildtype is shown in black. Pie charts are arranged in the order of presentation in the Results section of this manuscript, in pairs of yolk sac and embryo for each conceptus. Panels B-E depict the morphology of the respective embryos to the right: B: 29N-A; C: 29N-F; D: 29N-E, and E: 29N-G1.

The relative proportions of allele modifications in sequences from each tissue are displayed in pie charts (Figure 2, Panel A). The wildtype allele constitutes the majority of sequence reads in two sample pairs, 29N-A and 29N-F. For 29N-A, yolk sac and embryo returned 74.9% and 82.7% wildtype sequences, respectively. The CRISPR-induced edit, a 1 base pair insertion, is present in 25% of the reads obtained from yolk sac, and 17.3% of reads from the embryo, respectively. The non-equal ratio of mutant to wildtype alleles is incompatible with heterozygosity, which -by definition- comprises a 1:1 ratio of two alleles. Instead, the data indicate that the initial modification was propagated only in a subpopulation of cells that make up a small proportion of both embryo and yolk sac. Then, the individual is a mosaic of predominantly wildtype cells and those that carry the modification. Whether the mutant allele in those cells is present in heterozygous or homozygous configuration cannot be determined from our data unless individual cells are genotyped. Retention of cells with a wildtype allele is only possible if the CRISPR edit happened after the single-cell stage; the high fraction of wildtype sequences -and by inference wildtype cells- in this individual suggests that the edit likely occurred even later. Consistent with a low proportion of mutant cells is the morphologically largely normal appearance of the embryo (Figure 2, Panel B).

In sample pair 29N-F, the proportion of wildtype sequences is 56.5% and 57.2% in yolk sac and embryo, respectively. The unequal ratio of mutant to wildtype alleles again suggests that the individual is a mosaic, although an additional mutant allele is detected at low abundance in the yolk sac (see discussion). Despite the appreciable content of wildtype alleles, the embryo of 29N-F is devoid of somites (Figure 2 Panel C), indicating that the CRISPR induced edit affected the mesodermal lineage precursors in particular in this individual.

We also here find multiple modified alleles in the same individual, specifically in sample pairs 29N-E and 29N-G1, which each contain two CRISPR-modified alleles and the wildtype allele. For sample pair 29N-E, yolk sac and embryo revealed 36.8% and 42.2% of sequences with the Δ11bp@-5 deletion, and 34.6% and 37.7% of sequences with the 1bp insertion at @1, in addition to 26.5% and 20.1% of wildtype sequences, respectively. Because heterozygosity for a mutant allele and wildtype would yield a higher representation of wildtype than is observed here, we have to conclude that the two mutant loci co-occur in the same cells, a condition termed “compound” homozygosity. Thus, this individual again is a mosaic, composed of cells that contain the two mutant alleles, and of wildtype cells. Based upon sequence representation, the proportion of mutant cells is approximately 75%, with only 25% wildtype cells, indicating that the mutations were induced at the two-cell stage, and that the mutant cell descendants subsequently gave rise to the greater share of embryonic cells. The embryo of this sample pair (Figure 2, Panel D) lacks somites, the neural tube is open all along the rostrocaudal axis, and at the primary closure initiation site, translucent bubbles are visible on either side of the wavy midline.

In sample pair 29N-G1, 59.1% of the yolk sac and 77.8% of the embryo sequences represent the largest deletion Δ1093bp@-2, while the 1bp insertion +1@1 appears only in 20.8% and 9.5% of yolk sac and embryo sequences, respectively. Intriguingly, these frequencies are closely mirrored in the proportions of 20.1% and 12.7% wildtype sequences in yolk sac and embryo, respectively, suggesting that the insertion co-occurs in heterozygosity with the wildtype allele in the same cells. Then, the deletion allele would be predicted to be in homozygous configuration, which would make up the vast majority of cells in the embryo, and still a large majority in the yolk sac. Consistent with the interpretation that mutant cells predominate in the embryo is its phenotype (Figure 2 Panel E): The entire posterior neural tube of the 29N-G1 embryo is open, and the somites in this region are very small. In the midsection of the embryo, somites seem to have normal size, but are misshapen, associated with a wavy and incompletely closed neural tube appearance.

Taken together, our results reveal CRISPR-induced gene edits at the T/brachyury locus that recapitulate mutations from our prior discovery in an independent experiment, and also produce distinct new mutations. In each mutant conceptus, the allele frequencies reveal unique genetic configurations, contributing to different cellular compositions of the embryos, all of which were mosaics, with distinctive consequences for the observed morphological phenotypes. Importantly, the results provide unequivocal evidence that mutant alleles that occur in the embryo are also represented in the corresponding yolk sac.

## Discussion

**Figure 3:**
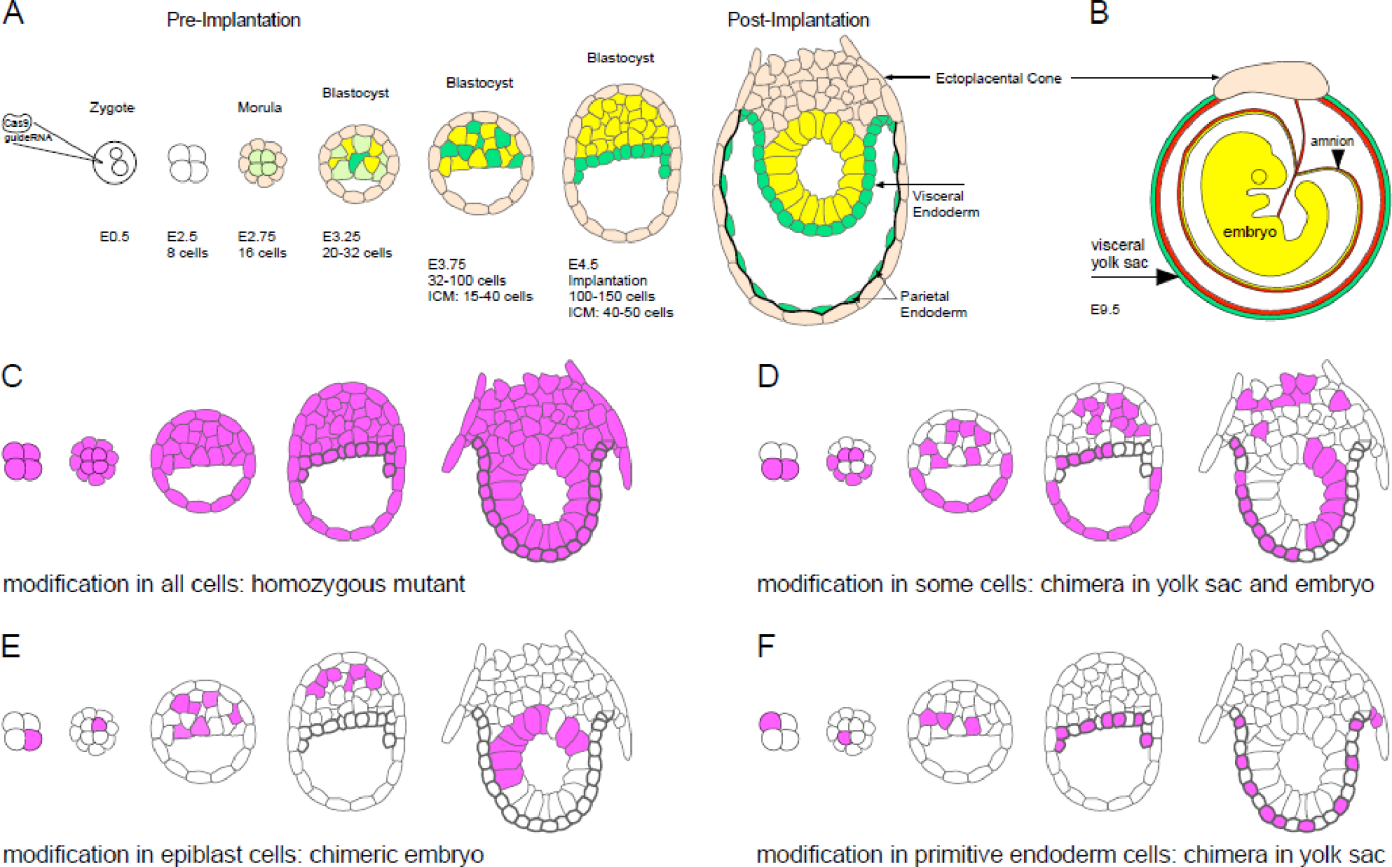
Scenarios for the segregation of CRISPR edits during cell lineage determination in the blastocyst Microinjection of Cas9 protein together with guide RNA targeted to the T/brachyury gene was performed in fertilized oocytes. Panel A depicts pre-implantation development (modified from ^21^): After the first cell division, the blastomeres are initially equivalent, until at the morula stage cells on the outside are fated to become trophectoderm (light orange), which produces extra-embryonic tissue such as the ectoplacental cone, and the inside cells are fated to become inner cell mass. Initially bipotential, (lime-green) they then are specified to give rise to the primitive endoderm (green) or epiblast (yellow) progenitors. After primitive endoderm precursors get sorted to face the blastocoel cavity, they give rise to the visceral endoderm (green), and contribute to the parietal and visceral yolk sac later. The embryo proper derives from progenitors in the epiblast (yellow). Panel B (modified from ^52^): By E9.5, the visceral yolk sac consists of an endoderm layer derived from primitive endoderm (green) and a mesodermal layer (red) derived from the embryo. This extraembryonic membrane is typically used for genotyping F0 conceptuses with genetic modifications. Panels C-F depict theoretically possible segregation of cells with mutant alleles after CRISPR editing; schematics were reduced for clarity (see Discussion for detailed considerations of each scenario).

We have previously shown that a wide spectrum of variant alleles can be generated by CRISPR edits at the T/brachyury gene in F0 progeny^11^. Having detected up to 7 distinct alleles, including wildtype, in the same individual, we reasoned that creation of these discrete events must have involved up to 7 cell divisions, during which the embryo could have grown to 128 cells (2^7^=128), the size of the blastocyst prior to implantation. *In vivo*, this process can take up to 3 days, during which the separate germ layers are specified (Figure 3, Panel A). Primitive endoderm progenitors are amongst the earliest cells specified in the preimplantation embryo^20,21^. They contribute to the visceral endoderm and the endodermal cell layer of the visceral yolk sac, which also consists of a layer of embryo-derived mesodermal cells (Figure 3 Panel B). The temporal coincidence of cell lineage segregation with multiple rounds of allele editing by CRISPR could potentially result in different allele composition of the embryo when compared to its associated yolk sac, with at least 4 principal scenarios possible.

For reasons of conceptual and visual simplicity, each scenario depicted in Figure 3 (Panels C-F) is elaborated with only one mutation: Scenario in Figure 3 Panel C: The CRISPR edit occurs before 4-cell stage. Regardless whether the edited cell is homo- or heterozygous for a mutant allele, all cells will be mutant, including trophectoderm derivatives. The predicted outcome is that yolk sac and embryo will have the same genotype. Scenario in Figure 3, Panel D: The CRISPR edit occurs in only some cells, other cells remain wildtype. The conceptus will be mosaic in yolk sac, embryo and trophectoderm derivatives. Regardless whether the edited cells are homo- or heterozygous for a mutant allele, yolk sac and embryo will have the same genotype. Scenario in Figure 3, Panel E: Only one blastomere undergoes a CRISPR edit, the other cells retain wildtype alleles. The CRISPR edited cell gets specified as epiblast, primitive endoderm cells are wildtype. Unless all epiblast cells derive from the mutant cells, the embryo will be a mosaic. Regardless whether the edited cells are homo- or heterozygous for a mutant allele, only the embryo will genotype as containing a mutant allele, and -if a mosaic- also wildtype alleles; the yolk sac will genotype as wildtype. Yolk sac genotyping alone will miss the F0 CRISPant embryo. Scenario in F: CRISPR editing occurs in a blastomere whose descendants are specified as primitive endoderm only; epiblast cells will remain wildtype. Regardless whether the edited cells are homo- or heterozygous for a mutant allele, only the yolk sac will genotype as containing mutant allele, and possibly wildtype; the embryo will genotype only as wildtype. In both scenarios E and F, if cells are present that harbor another distinct mutant allele, they could be segregated into the epiblast or yolk sac according to any of scenarios D, E, and F.

In light of several theoretically possible scenarios, this study therefore sought to determine whether yolk sac genotyping appropriately reflects embryonic CRISPR-induced genomic editing events. We detected CRISPR edits in 4 pairs of embryo and corresponding yolk sac. Interestingly, 3 of the 6 discrete CRISPR modifications are identical to those found in multiple individuals in our prior report^11^ (+1bp@1; Δ1bp@-1, and Δ11bp@-5), but we here also identified 3 new distinct alleles (Δ8bp@-4; Δ119bp@-68; and Δ1093bp@-2). In contrast to our previous experience^11^, where all 39 out of 39 analyzed conceptuses were CRISPants with mutant alleles, we here detected modifications in only 4 out of 10 conceptuses. This could be due to a different batch of Cas9 enzyme, or the occurrence of deletions that would not be detected in our genotyping assay, e.g. if one of the binding sites for the amplification primers was deleted. This would be the case if deletions extended over 3.3 kb from theta in either direction. Large deletions have been reported in the literature^9,22–27^ for cultured cells and for whole organisms, as much many as over 0.5 Mbps were unintentionally deleted- ^22^ in a transgenic approach, similar to the one pursued in this study. Yet, the systematic application of long-read sequencing to CRISPR genotyping has been pursued only by a few research groups so far^8,10,11,23,28–30^, and the present study; conventional CRISPR genotyping overwhelmingly uses techniques that survey for edits only in a narrow envelope around theta^31–34^, and therefore miss events that create large deletions, inversions and other structural mutations. Nevertheless, the sample number of 4 CRISPants is sufficient to draw valid conclusions, according to the long-standing convention in the transgenic mouse field that three independent genetic events yielding the same outcome establish the observed outcome as caused by the genetic manipulation^35^, as opposed to some random manifestation, which would not be expected to occur consistently among multiple individuals.

Single-molecule sequencing in this study not only ascertained the particular CRISPR-mediated edits, but also yielded information on the relative representation of these edits among all recovered DNA sequences of the T/brachyury locus. The proportions of discrete alleles allow us to predict particular genetic configurations and cellular composition for each conceptus, all of which must have been mosaics. This is consistent with our earlier study^11^, in which at least 34 out of 39 individuals were mosaics, predicted from allele abundance in the yolk sac. Ascertainment of cellular composition and genetic configuration is particularly important in experimental approaches that have to investigate the F0 generation^3,4,36–43^, such as e.g. when permanent lines of mutant animals cannot be derived due to embryonic or perinatal lethality after mutation, or e.g. when multiple loci are targeted simultaneously that would segregate with breeding. Where live offspring result and can be propagated, the next generation individuals will contain only those alleles transmitted through the germline, and therefore all of their cells will have a uniform genotype^35^.

With regard to our overarching question, namely to what extent yolk sac genotyping produces an appropriate reflection of embryonic genotype, our results provide unequivocal evidence: all discrete alleles detected by DNA sequencing in the embryo were also, in all cases, present in the yolk sac. This is consistent with the presence of mesodermal cells in the visceral yolk sac, which derive from the embryo and therefore would be expected to contain alleles that are also edited in the embryo (Figure 3 Panel B). Thus, we conclude that our results strongly support the scenarios depicted in Figure 3, Panels C and D, where yolk sac genotyping faithfully reports all alleles that comprise the embryonic genotype. Although conceptuses comprised entirely of mutant cells in embryonic and extraembryonic tissues (as shown in Figure 3, Panel C) were not recovered here, they constituted around 30% of cases in our previous study^11^, based on yolk sac genotyping. The hypothetical possibility where the embryo contains alleles not found in the yolk sac (shown in Figure 3, Panel E) could only occur if the embryo is a mosaic, in which the mesoderm that contributed to the yolk sac is made up of only wildtype cells. Then, the yolk sac would genotype as wildtype, and any embryonic CRISPR modified alleles would be missed. However, as mesoderm arises post-implantation -during gastrulation^44–46^- from a comparatively large number of progenitor cells, the scenario that they would all be wildtype is highly unlikely.

On the other hand, the potential outcome where yolk sac might contain alleles not present in the embryo (Figure 3, Panel F), is represented in one of our cases: in conceptus 29N-F, the allele with an 8bp deletion Δ8bp@-4 from ϴ is only found among yolk sac sequences, and not in the embryo. Thus, it must have arisen in a precursor cell that specifically contributed only to the yolk sac, and the very low representation of this allele, at 4.6% of all sequences for this yolk sac sample, indicates that further cell proliferation was minimal compared to other cell lineages in the yolk sac. Such type of event appears to be rather uncommon: in our earlier study, very low representation of a mutant allele (<5%) was observed in only 4 out of 39 yolk sac samples^11^. In cases of low abundance of variant alleles then, subsequent genotyping of embryonic DNA would be warranted to obtain an accurate determination of embryonic CRISPR-induced edits and mutations, and for interpretation of phenotypes in mosaic F0 individuals^6^.

Finally, we show that the CRISPR editing strategy produces morphological anomalies in the manipulated embryo, dependent on the proportion of cells with mutant alleles within the mosaics we recovered in this study. Compared to chimeras between T-mutant and wildtype cells generated by conventional methods^15^, and compared to the classical T/brachyury mutant^47,48^, our mosaic T-edited CRISPants display the same spectrum of developmental defects, including absence of somites or misshapen somites, abnormal development in the posterior region, open neural tube, and kinked neural tube. Thus, by targeting the T/brachyury gene, we were able to make evident the yolk sac-embryo correlations of genomic edits, and demonstrate their relevance in body structures whose development is dependent on mesoderm generated during gastrulation and on the subsequent proper migration of mesodermal derivatives^49,50^.

## Conclusions

We recovered CRISPR/Cas9-induced mutations at the T/brachyury gene in F0 CRISPant mouse embryos, and showed that their associated visceral yolk sacs contained mutant alleles that correctly reflect embryonic genotype. This is particularly relevant for diagnosis and interpretation of morphological anomalies that arise as a consequence of CRISPR modifications, when only the F0 generation is available due to subsequent peri-natal lethality caused by the induced mutations.

Furthermore, the abundance of mutant alleles detected by single-strand long-range sequencing in this and our prior study indicated that all CRISPant embryos were mosaics of wildtype and mutant cells, in different configurations of hetero- and homozygosity. We discuss these results in light of cell specification processes in pre-implantation development, and highlight a particular scenario in which -in addition to the routine genotyping of yolk sac tissue- embryonic tissue should also be tested to achieve accurate genotype determination.

Our discovery of new edits that were not detected in previous studies, and the high prevalence of mosaicism observed in our CRISPants, add to the growing literature^51^ on limitations of CRISPR/Cas9 genome editing *in vivo*.

## Figures and Tables

**Table 1:** Alleles according to CRISPResso Analysis

Sample name	INDEL 1	in %	INDEL 2	in %	% wildtype
N79M33–1 (control)					100.0
N79M33–2 (control)					100.0
					
26N-A-VYS					100.0
26N-A-E					100.0
					
26N-B-VYS					100.0
26N-B-E					100.0
					
29N-A-VYS	ins +1bp@1	25.0			74.9
29N-A-E	ins +1bp@1	17.3			82.7
					
29N-B-VYS					100.0
29N-B-E					100.0
					
29N-C-VYS					100.0
29N-C-E					100.0
					
29N-D-VYS					100.0
29N-D-E					100.0
					
29N-E-VYS	Δ11bp@-5	36.8	ins +1bp@1	34.6	26.5
29N-E-E	Δ11bp@-5	42.2	ins +1bp@1	37.7	20.1
					
29N-F-VYS	Δ119bp@-68	39.0	Δ8bp@-4	4.6	56.5
29N-F-E	Δ119bp@-68	42.8			57.2
					
29N-G1-VYS	Δ1093bp@-2	59.1	Δ1bp@1	20.8	20.1
29N-G1-E	Δ1093bp@-2	77.8	Δ1bp@1	9.5	12.7
					
29N-G2-VYS					100.0
29N-G2-E					100.0

Genotyping of yolk sac and embryo genomic DNA for CRISPR-induced mutations was performed by single-molecule long-read sequencing (Oxford Nanopore Technologies) of PCR amplicons generated by primers that spanned 6.6 kb of the wildtype sequence^11^. Variants were identified by CRISPResso2 analysis. Sample pairs of visceral yolk sac (extension -VYS) and embryo (extension -E) are listed in rows close together, and came from two surrogate pregnancies. The first two rows list DNA controls that came from wildtype FVB/N embryos. The representation of variant sequences is given in % of all sequences for a given sample that had passed quality criteria and revealed an insertion or deletion, in short INDEL. Six distinct modified alleles were found, as well as wildtype. The length of each insertion (+) or deletion (Δ) is listed relative to (@) the 5’ breakpoint upstream of the guide-RNA targeted cut site theta ϴ.
